# Waste Valorization towards Industrial Products through Chemo- and Enzymatic- Hydrolysis

**DOI:** 10.1080/21655979.2023.2184480

**Published:** 2023-06-28

**Authors:** Daniel Szopa, Dawid Skrzypczak, Grzegorz Izydorczyk, Katarzyna Chojnacka, Konstantinos Moustakas, Anna Witek-Krowiak

**Affiliations:** a Department of Advanced Material Technologies, Faculty of Chemistry, Wrocław University of Science and Technology, Wroclaw, Poland; b School of Chemical Engineering, National Technical University of Athens, Athens, Greece

**Keywords:** Hydrolysis, enzymatic, chemical, extraction, fertilizer, amino acid

## Abstract

This paper reviews the scientific literature on the latest technologies for treating waste by chemical hydrolysis, enzymatic hydrolysis and supporting processes. Particular attention is focused on wastes of biological origin, especially high-protein materials and those containing fats and sugars, as valuable components can be extracted from these recyclables to produce plant growth-stimulating compounds and animal feed, chemicals, biofuels or biopolymers. The wastes with the greatest potential were identified and the legislative regulations related to their processing were discussed. Chemical and enzymatic hydrolysis were compared and their main applications directions and important process parameters were indicated, as well as the need to optimize them in order to increase the efficiency of extraction of valuable components.

## GRAPHICAL ABSTRACT

**Figure:**
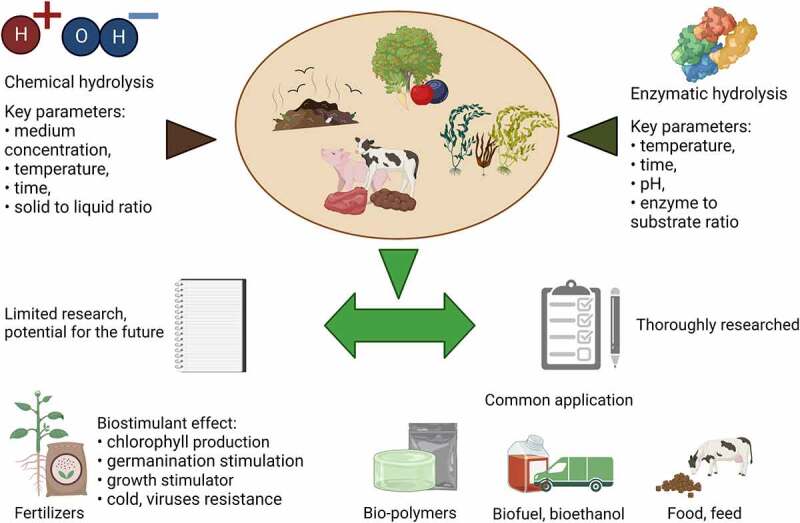

## 1. Introduction

Currently, the world population is estimated to have reached 7.5 billion, growing continuously, and will reach 8 billion in the next year [1]. The ever-growing population corresponds to the constant demand for food and the need for agricultural intensification. At the same time, increasing amounts of waste are generated, especially biowaste, which requires management [2]. Biowaste is rich in valuable elements that might be successfully retrieved using appropriate techniques, according to the circular economy. The circular economy is designed to minimize or eliminate nonrenewable feedstocks and maximize the recovery of materials and energy from waste, thus closing the loop. This practice also reduces greenhouse gas emissions [3], benefiting the environment and achieving sustainability goals. Ideally, zero-waste and zero-emission production would be the ideal solution [4]. Biowaste is a broad group of carbon- and nitrogen-rich materials that are reservoirs of proteins [5], carbohydrates, and fats. These compounds are the feedstock for many conversion processes that can produce valuable products (bioactive compounds, biofuels, biopolymers, fertilizer compounds) and energy (biogas) [6]. The most common processes for the treatment of biowaste include biological processes (anaerobic digestion, composting, enzymatic processes), chemical processes (acid and alkaline hydrolysis), or thermal processes (pyrolysis, gasification).

Organic compounds can be hydrolyzed, resulting in valuable products such as peptides and amino acids, sugars, or lipids. Hydrolysis is carried out using acids or bases, often at elevated temperatures. Enzymatic hydrolysis uses biologically active protein molecules with high enzymatic activity [7]. Hydrolysis occurring under subcritical [8] and supercritical [9] conditions could also be applied. Hydrolysis may be intensified with additional treatments such as ultrasound/cavitation [10], microwaves, or increased pressure. The recovery of valuable components from waste material by chemical hydrolysis is the main concern of the review. This process has the potential for the reduction of unit operations and for the separation of selected components. Acid or alkaline hydrolysis of waste high-protein materials yields hydrolyzates containing peptides and amino acids or separated proteins (e.g. collagen). It is possible to valorize waste from various industries such as leather [11], hairdressing [12], meat processing [13] or animal production [14] in this way. The products of protein hydrolysis may be successfully utilized in medicine, the food industry, the pharmaceutical industry or agriculture. Chemical hydrolysis of carbohydrates can be used as a method to recycle various textiles [15], lignocellulosic materials [16], food waste [17] to sugars and other active components. Hydrolysis of fats has the potential to produce fatty acids as feedstock for chemicals [18] or biofuels [19].

The aim of the review is to indicate the main applications of chemical and enzymatic hydrolysis for waste valorization. A critical analysis of the processes discussed has been made, which concludes with a list of improvements for the extraction of amino acids. This is essential to valorize the usage of hydrolysis for agricultural applications. The key inquiry is whether chemical hydrolysis can be an effective way to create biostimulants from high-protein materials. In this case, the process was compared with enzymatic hydrolysis, which is the most effective process for this purpose. The evaluation identifies modifications and solutions from other chemical hydrolysis applications that can be utilized to improve the extraction of amino acids. Research in this field is essential due to severe process condition, which degradate peptide chains.

## 2. Biowaste as a feedstock for chemical hydrolysis

The usefulness of animal and plant waste materials for various applications is highly dependent on their elemental composition. Nowadays, a lot of studies has been done on the possibility of retrieving amino acids that stimulate plant growth. The main goal of scientists is to obtain the highest possible degree of amino acid recovery. However, inexpensive methods lead to excessive degradation of peptide chains [20], primarily because of harsh parameters of conducting processes such as temperature, pressure and high concentration of extraction medium [21]. This tendency leads to research in process modification, e.g. chemical hydrolysis, which is extended by introducing pretreatment of the material or indirect processes that reduce the extraction conditions.

The main amino acids indicating a favorable effect on plant growth are Ala, Glu, Lys, and Ser, which are responsible for most of the stimulatory functions of plants, particularly in the early stages of development [22]. Most animal-derived materials contain high amounts of amino acids. Their overall nutritional composition is superior to the case of plant waste because they consist of more than 1.0 g/100 g of protein for each essential amino acid. In the initial stage of plant life, the key is to supply amino acids that promote seed germination, that is, Asp, Glu, Lys, Met, Phe, Thr [22]. Table 1 contains information on the amino acid profile and NPK content of various types of waste. These materials are often treated by chemical or enzymatic hydrolysis for their management issues [45,46]. Germination can be supported by amino acids from animal waste such as shrimp, slaughterhouse, wool, poultry litter, and biomass such as microalgae. All the mentioned wastes have an elevated content of crucial amino acids above 2.0 g/100 g of protein, while some of them (Asp, Lys, Glu) reach values above 10 g/100 g of proteins, which indicates a high potential for their use as biostimulant material. These wastes also contain large amounts of Ala, responsible for stimulating hormonal metabolism, chlorophyll production, and resistance to viruses and temperature changes [13,22]. Supplementing plants with Ala is crucial for survival in harsh soil conditions, variable weather, and hostile environments. The most considerable amounts found in a slaughterhouse, poultry waste, and microalgae are 11.45, 9.05, and 7.04 g/100 g of protein, respectively. Ala supplementation should help in the initial assimilation of the plant into the new environment. In the next stage of plant development, it is important to supplement Gly and Glu, mainly due to the stimulation of the chlorophyll production, which is substantial for light absorption and photosynthesis, which supports the overall development of the plant and is a reserve of organic nitrogen needed for the synthesis of other amino acids [22]. Together with Lys, Cys and His, both amino acids constitute a chelating agent. The role of amino acids in plant metabolism extends to many other functions, although a major concern is the large amount of energy that plants must use to synthesize them [47]. Therefore, their supplementation in the form of biostimulants is crucial.

**Table 1.** NPK content and amino acid profile of waste materials.

Type of waste	Amino-acid profile [%]	Nutrients profile [%]	References
Cereal grains	Ala − 0.74; Arg − 0.42; Asp − 0.68 Cys − 0.20; Gly − 0.36; His − 0.25; Iso − 0.39; Leu − 1.12; Lys − 0.27; Met − 0.23; Phe − 0.50; Pro − 0.73; Ser − 0.47; Thr − 0.37; Val − 0.52;	N − 1.47 P − 0.25 K − 1.29	[23,24]
Dry poultry waste	Arg − 2.30; Asp − 5.44; Glu − 6.46;Cys − 7.56; Gly; 1.58; Tyr − 3.60; Val − 2.68; Lys − 2.74; Met − 1.75; Ser − 1.99; His − 1.29; Thr − 2.28; Leu − 3.21; Phe − 2.04; Ala − 9.05; Iso − 2.10	N − 22.76 P − 2.65	[25]
Ginger fiber	Ala − 1.74; Arg − 3.90; Ast − 1.55; Cys − 0.38; Gln − 0.84; Gly − 0.55; His − 0.87; Iso − 1.97; Leu − 2.03; Lys − 1.84; Met − 0.55; Phe − 2.05; Ser − 1.96; Thr − 1.92; Val − 1.08	P − 4.2·10^−5^ K − 9.8·10^−5^	[26,27]
Microalgae	Ala − 7.04; Arg − 10.32; Asp − 12.16; Cys − 1.9; Glu − 14.64; Gly − 6.28; His − 1.76; Iso − 4.82; Leu − 8.86; Lys − 6.64; Met − 2.96; Phe − 5.28; Pro − 6.30; Ser − 5.02; Thr − 4.74; Trp − 2.64; Tyr − 3.84; Val − 5.74	N − 2.88 P − 0.80 K − 0.20	[28,29]
Municipal solid waste	Ala − 0.0036; Arg − 0.0022; Asp − 0.0045; Cys − 0.0005; Glu − 0.0057; Gly − 0.0036; His − 0.0006; Iso − 0.0020; Leu − 0.0038; Lys − 0.0016; Met − 0.0007; Phe − 0.0022; Pro − 0.0020; Ser − 0.0019; Thr − 0.0020; Tyr − 0.0016; Val − 0.0022	N − 1.10 P − 0.32 K − 1.07	[30,31]
Oak extract	Ala − 0.0012; Arg − 0.0043; Asp − 0.0035; Gaba − 0.0004; Gln − 0.0012; Glu − 0.0022; His − 0.0005; Ile − 0.0003; Leu − 0.0006; Lys − 0.0005; Phe − 0.0004; Pro − 0.013; Thr − 0.0009; Trp − 0.0002; Tyr − 0.0005; Val − 0.0006	N − 0.8 P − 0.35 K − 0.15	[32,33]
Orange peel waste	Arg − 0.59; Asn − 0.72; Asp − 4.14; Cit − 0.09; Cys − 0.08; Gln − 0.29; Glu − 1.00; Gly − 2.30; His − 0.73; Iso − 0.29; Leu − 0.73; Lys − 0.91; Met − 0.17; Orn − 1.24; Phe − 0.36; Pro − 6.15; Ser − 4.71; Thr − 0.75; Try − 0.10; Tyr − 0.49; Val − 0.85	N − 1.5 P − 0.7 K − 2.3	[34,35]
Pineapple by-products	Asn − 0.008; Cys − 0.00025; Gln − 0.014; Glu − 0.0023; Ser − 0.01; Thr − 0.0012; Tyr − 0.00025; Val − 0.00025	N − 1.08 P − 0.083 K − 1.14	[36,37]
Shrimp waste	Ala − 2.9; Arg − 3.56; Asp − 2.9; Cys − 2.16; Glu − 9.66; Gly − 2.73; His − 4.71; Iso − 1.8; Leu − 3.76; Lys − 4.89; Met − 2.77; Phe − 2.81; Pro − 2.62; Ser − 3.7; Ser − 3.70; Thr − 3.35; Tyr − 3.79; Tyr − 3.79; Val − 4.86	N − 1.08 P − 0.047 K − 0.08	[38]
Slaughterhouse waste	Ala − 11.45; Arg − 3.61; Asp − 11.81; Glu − 9.29; Gly − 5.04; His − 5.85; Iso − 0.34; Leu − 13.80; Lys − 10.01; Phe − 8.08; Ser − 5.95; Thr − 5.53; Val − 5.31	N − 4.94 P − 0.16 K − 0.11	[39,40]
Spent coffee grounds	Ala − 0.53; Arg − 0.02; Asp − 0.77; Cys − 0.02; Glu − 1.82; Gly − 0.67; His − 0.18; Iso − 0.48; Leu − 1.04; Lys − 0.13; Met − 0.13; Phe − 0.67; Pro − 0.53; Ser − 0.08; Tau − 0.13; Thr − 0.18; Try − 0.08; Tyr − 0.32; Val − 0.69	N − 2.37 P − 0.89 K − 3.72	[41,42]
Vine shoots extract	Ala − 0.021; Arg − 0.27; Asn − 0.0052; Asp − 0.015; Cit − 0.0020; Gaba − 0.049; Gln − 0.11; Glu − 0.047; His − 0.0043; Ile − 0.0002; Leu − 0.0019; Lys − 0.0023; Phe − 0.0009; Pro − 0.025; Ser − 0.0067; Thr − 0.0063; Trp − 0.0033; Tyr − 0.0005; Val − 0.0027	-	[32]
Waste human hair	Ala − 0.12; Asp − 0.097; Cys − 0.030; Glu − 0.089; Gly − 0.039; Ile − 0.046; Leu − 0.16; Lys − 0.078; Phe − 0.029; Pro − 0.11; Ser − 0.018; Thr − 0.026; Tyr − 0.059; Val − 0.083	N − 12-16 P − 0 K − 0	[33,43]
Wool	Ala − 3.79; Arg − 9.17; Asp − 6.41; Cys − 11.33; Glu − 13.13; Gly − 5.27; His − 0.97; Iso − 3.73; Leu − 7.72; Lys − 3.12; Met − 0.54; Phe − 3.44; Pro − 6.41; Ser − 7.33; Thr − 6.48; Tyr − 4.17; Val − 5.87	N − 16.82 P − 0.020	[44]

**Figure 1.**
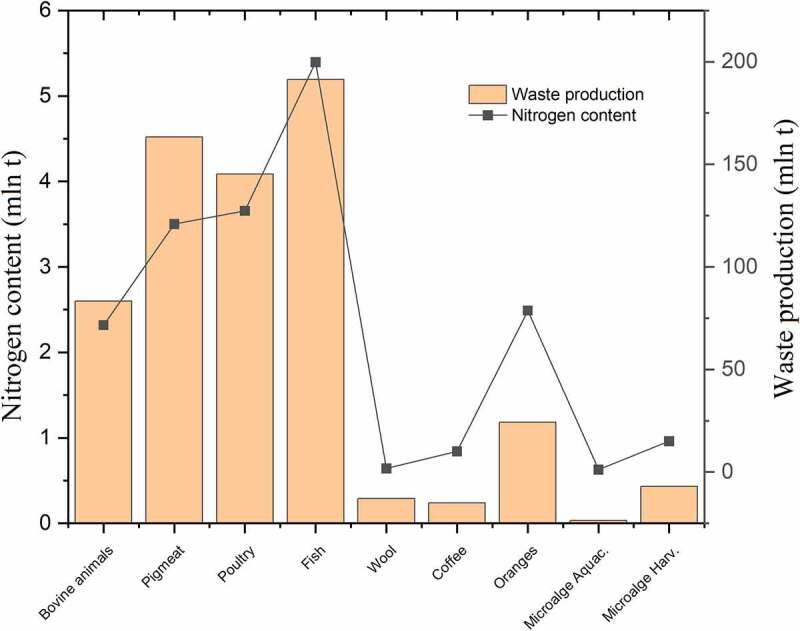
Waste production with a rich amino acid profile. Waste nitrogen sources are shown along with the amount (in Mg) of waste nitrogen they contain [48–62]. .

With the increasing population and demand for food products, animal and plant waste requires new disposal and management methods. The production of these wastes is significant, as seen in Figure 1.

The amount of nitrogen in the waste presented in Figure 1 indicates the content of amino acids. Research over the years has shown that a total amount of crude protein, can be assumed by a conversion factor of 6.25 · nitrogen content [63]. This resulted in approximate value, where about 16% of the amino nitrogen is the weight of the proteins [64]. This information allows for an estimation of the waste potential without the need for an amino acid content analysis. It is especially useful because information on the exact amount of amino acids for different wastes is not available. In recent years, methods of extracting amino acids have been studied for biostimulating purposes, along with the creation of comprehensive forms of fertilization in terms of NPK composition. The mentioned wastes contain significant amounts of amino acids and they also have important nitrogen content reaching more than 20%, that is, poultry waste [25]. Although this causes a disturbance of the macronutrient ratio, mineral additives or other waste added to the mixed fertilizer should be balanced in the composition of the NPK. The issue of balancing the NPK profile looks more promising for plant waste, which mostly has similar macronutrient content, but the total amount does not exceed 4% and the amino acid profile itself is limited. Only cereal grains, ginger fiber and orange peel waste can obtain single amino acids such as Asp, Pro, Ser, and Arg. Orange peel waste has the best balance of NPK and a significant content of amino acids and can be a source for producing biostimulants of plant origin. The highest potential of all mentioned plant materials has microalgae, which are applied on a large scale in pharmaceuticals, cosmetology and the food industry [59]. They are also utilized for biomass production and biofuel production [65]. The wide range of their usage causes their production to increase over the years constantly. For these reasons, the methods of utilization of raw materials that are easy to modify and produce seem to be a key issue [66]. Microalgae have a rich amino acid profile, containing Ala, Arg, Asp, Glu, which is 7.04, 10.32, 12.16, and 14.64 g/100 g of protein. The remaining have an average value of 4.77 g/100 g of protein, indicating a significant extraction potential [28]. The other agricultural waste mentioned in Table 1 contains negligible amounts of amino acids below 0.1 g/100 g of protein; therefore, their management in terms of amino acid extraction is redundant [32]. They can be used to supplement potassium and phosphorus in high-protein waste with a low content of the macroelements mentioned above. The above dependence occurs in municipal waste, which can be a particular source of potassium to supplement amino acid compositions. Their significant amount allows for a large source of renewable potassium and phosphorus. The main issue with the waste mentioned is the notable diversification based on the region and disposal methods [30,31]. The composition of the waste may vary significantly and limits its application. However, it is an important issue to evaluate in terms of fertilizer-biostimulant for achieving sustainable development goals.

## 3. Legal aspects

According to the waste hierarchy, the first step should be to reduce the production of waste. If this is not possible, recovery processes should be implemented, especially through material recycling. The path of waste management, including bio-waste, is presented in Directive 2008/98/EC of the European Parliament and of the Council of 19 November 2008 on waste and repealing certain Directives and supporting documents [67]. According to the legal definition, bio-waste is portrayed as biodegradable waste, including green waste from urban areas, waste from food production and processing, including out-of-date food and waste from mass catering facilities. The law clearly indicates that bio-waste must be isolated and recycled at source or selectively collected and not blended with other types of waste, especially hazardous or non-biodegradable waste. This makes it much easier to further process or utilize them.

**Figure 2.**
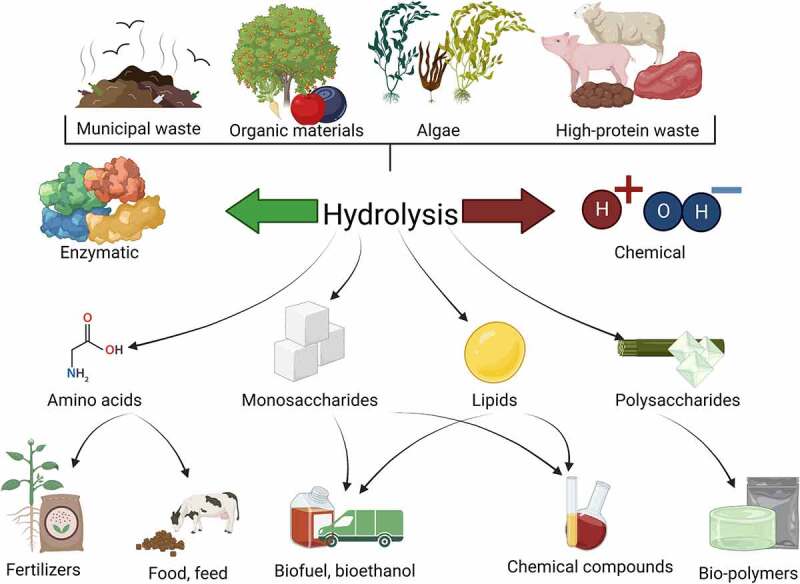
Scheme of the potential of waste materials and their management methods.

In principle, the state of knowledge, science and technology allows waste to be managed or treated in many ways. The Waste Framework Law indicates that EU Member States should primarily encourage the domestic treatment of bio-waste through composting or digestion and support the domestic use of materials resulting from digestion and composting [67]. Statutory guidance on bio-waste management options does not preclude other methods. In order to prioritize material recycling over energy recycling, methods other than incineration or co-incineration should be implemented. Hydrolysis – acid, alkaline or enzymatic – is widely described in the literature. Waste management options along with commonly utilized methods of disposal are presented in Figure 2.

Chemical hydrolysis can also be applied to other wastes that do not fall under the definition of bio-waste. It should be noted, however, that processing of such waste is often impossible or very difficult – for example, animal waste. The basic legal act – Regulation (EC) No 1069/2009 of the European Parliament and of the Council of 21 October 2009 laying down health rules as regards animal by-products and derived products not intended for human consumption and repealing Regulation (EC) No 1774/2002 (Animal by-products Regulation) - divides animal waste into three categories [68]. Animal by-products, by nature, contain animal tissue that can be easily hydrolyzed. Unfortunately, waste from animal breeding and processing classified as category 1 animal by-products have to be incinerated or co-incinerated because they are considered hazardous. This category includes, for example, animals suspected of carrying TSE or BSE, pets, circus animals, wild animals or animals which have been the subject of illegal treatment or testing, as well as animal by-products from the treatment of wastewater from animal farms and waste from international shipments. These materials are considered as carriers of diseases, often with high infectious potential or unknown diseases. Only thermal treatment guarantees complete inactivation of disease-causing pathogens. Analyzing the European Waste Catalog, we can find many wastes that can be hydrolyzed, but their processing by this method can be ineffective or insufficient. For example, waste from the tanning industry may contain high concentrations of chromium. Hydrolysis will decompose the material, but there will still be a need to remove the chromium before further use of the hydrolyzate [67]DirectiveD.

The utilization of hydrolysis for the treatment of bio-waste (by definition) and other biodegradable wastes is legally allowed and is an alternative to the generally applicable management methods such as composting, digestion, incineration or co-incineration. However, before management is undertaken, the waste should be clearly assigned to an entry in the European Waste Catalogue to exclude the mismanagement of hazardous waste. This approach guarantees the possibility of using bio-waste hydrolyzates in agriculture, e.g. as bio-stimulants or fertilizers. However, it should be noted that the final product used in plant cultivation, which is a hydrolyzate or the hydrolyzate is part of it, has to meet the quality requirements for agrochemicals by the law. Conditions for the use of biowaste in fertilizer production as well as fertilizer quality requirements (minimum nutrient content, maximum heavy metal content, maximum bacterial content, etc.) are described in Regulation (EU) 2019/1009 of the European Parliament and of the Council of 5 June 2019 laying down rules on the making available on the market of EU fertilizing products and amending Regulations (EC) No 1069/2009 and (EC) No 1107/2009 and repealing Regulation (EC) No 2003/2003 [67,69].

## 4. Chemical hydrolysis

Chemical hydrolysis is a relatively slightly researched method of organic waste valorization to extract desired components from biowaste. It is mainly utilized to extract polysaccharides to obtain nanoparticles. This approach allows the use of waste materials to create specialized components in a variety of industries. The production of biofuels has also received considerable attention due to the significant decomposition of glycosidic chains, i.e. obtaining simple sugars and the extraction of fats from high lipid media. However, because of the harsh conditions that lead to chemical hydrolysis, the recovery of amino acids seems to be not very effective. Mainly as a result of excessive degradation of peptide chains. For this reason, enzymatic hydrolysis is more commonly utilized. Depending on the extraction contact time and the concentration of the extractive solution, one or two phases are obtained in chemical hydrolysis and could be used in various applications.

### 4.1. Industrial applications

Chemical hydrolysis is often utilized to obtain polymers of saccharides and lignin, e.g. from food waste. Acid hydrolysis reduces complex sugars to reducing sugars, also breaking down peptide chains [70]. Some publications have shown that treatment with inorganic solvents can cause the extraction of macronutrients such as nitrogen, phosphorus and potassium. The main advantage of chemical hydrolysis is the high degree of solubilization of high-protein materials, up to 80% [71]. In the article by Du et al., (2018) [72], the solid phase was obtained due to the hydrolysis of food waste compost at a temperature of 60–100°C. It indicated a better macronutrient composition than standard compost made of the same type of materials, especially nitrogen content. The fertilizer was evaluated in field tests on red peppers and beans. The increase in yield was 90% [73] 77% (soluble proteins in leaves), 226% (soluble proteins in roots) [74]. The fertilizer contained primarily soluble saccharide and lignin-like polymers [72]. Lignin is a polysaccharide responsible for improving plant cell wall rigidity and mineral exchange by the fibrovascular bundle [75]. This indicates the potential of chemical hydrolysis as a simple way of managing waste for fertilization purposes. The studies mentioned above confirmed the potential of the process in the management of agricultural and food waste, although the growing demand for high-protein products requires scientists to expand their research in this area. More attention is paid to the possibility of recovering amino acids that have stimulating properties for plants, which directly affects the abundance of crops [76]. Few scientific publications describe the use of chemical hydrolysis to extract amino acids and the possibility of their use in plants as a biostimulant of growth. Izydorczyk et al., (2022) [13] indicated that it is possible to obtain a liquid fertilizer containing amino acids (0.5%) by acid hydrolysis with H_2_SO_4_ and H_3_PO_4_. A rich microelement profile was obtained due to the management of waste from the slaughter industry with an increase in micronutrient density of 31.2% Cu; 10.5% Mn; 33.9% Zn. Due to its biostimulating properties and its high nitrogen content, the fertilizer itself caused an increase in stem length (8.2%); chlorophyll content (27.0%) compared to the control sample. To verify the potential of both plant and animal waste, 39 waste (food streams) were tested for the amino acids. The significant potential of the amino acid profile of the studied groups was demonstrated [5]. The utilization of anaerobic digestion, composting, pyrolysis, etc., is considered, which indicates the need to optimize the methods and look for effective methods of managing the mentioned waste [45,46,77]. The article by Prandi et al., (2019) [5] also showed that waste containing low nutritional properties, eg, as animal feed, can be used for fertilization (orange peels, berry waste), and in some cases (hard cheese, pumpkin kernel cake) recovery of the total amount of crude protein is over 50%. The potential of amino acid recovery methods was indicated in the work by Xu et al. (2018);Wei et al. (2019) [78,79], where the usage of protein hydrolyzates in the form of feed significantly increased the growth and composition of farmed fish. An interesting application of the hydrolyzates is to utilize them as replacements for fungicides that simultaneously support the plant’s development. The paper by Jindřichová et al., (2018) [80] presented that the utilization of alkaline hydrolysis of food waste and garden waste allows limiting the growth of *L.maculans* by 42–56% (applied to leaves) and 31–37% (applied to roots). The results points the need to extend research in the field of the use of hydrolyzates as plant protection products.

### 4.2. Process parameters

Hydrolysis is used to valorize waste materials to produce useful products for various industrial sectors. In most cases, it is one of many unit processes leading to preparing a target product or semi-product. In the literature, in most cases, hydrolysis is used to obtain sugars in the form of cellulose nanocrystals [81] and nanocellulose fibers [82], which are used to produce high-quality biomaterials, applied in the biomedical, food or electronics sectors [83]. Hydrolysis to monosaccharides allows the diversion of glucose, fructose or xylose to fermentation processes. The main direction of using such intermediates is the production of biofuels (bioethanol) [84–86]. To a lesser extent, research focuses on the direct valorization of fatty acids, also used for biofuel production [19]. The hydrolysis of high-protein waste breaks down proteins into amino acids and short peptides. Due to their plant growth-stimulating properties, they are applied to produce specialized fertilizers [8,13]. This type of waste is also processed into collagen [87] or keratin [88], the directions of its management are connected with the production of biomaterials [88], but also food products [87].

Obtaining the target product is directly related to the appropriate material preparation in the processes preceding hydrolysis, the selection of the process parameters, and the 2 selection of subsequent unit processes after treatment with acids/hydroxides [18]. The selection of process parameters is directly related to the efficiency of the process [82]. Much attention is paid to parameters such as hydrolysis medium and its concentration, process temperature, contact time of the raw material with the acid/hydroxide and the ratio of waste material to medium (S:L) (Table 2). Additional substances are, in some cases, used to increase efficiency while reducing contact time or hydrolysis temperature [81]. Determining the efficiency of the process is closely related to the expected product of the process – e.g. crystallinity index [96], content of monosaccharides [90], degree of hydrolysis of proteins to amino acids [88] or recovery of lipids and carotenoids [100]. In some studies, optimization of process conditions is performed using experimental planning methods and mathematical modeling (e.g. Response Surface Methodology), which reduces the number of experiments and testing time [93].

**Table 2.** Chemical hydrolysis of waste materials as a valorization process to useful products.

Material	Product	Medium	S:L ratio	Temperature	Time	Effect	Application	References
Waste human hair	Amino acids	KOH:NaOH (14:1 ratio) (0.5, 1, 2, 3, 4% m/m) → Acoustic and hydrodynamic cavitation	1g:100ml	30°C	24h	n.d. (identification of amino acids)	Amino acids fertilizer	[12]
Waste production with a rich amino acid profile. Waste nitrogen sources	Amino acids	H_2_SO_4_ (96% m/m)+ H_3_PO_4_ (85% m/m) (2:1 ratio)	1g:1g	40°C	24h	Amino acid content: 0.5% m/m	Amino acids fertilizer	[13]
Poultry waste (chicken intestine)	Amino acids	H_2_SO_4_ (0.02% m/m) (supercritical water)	n.d.	260°C	28min	Efficiency: 11.49%	Amino acids fertilizers	[8]
Orange peel waste	Reducing sugars	H_2_SO_4_ (0.5, 1, 1.4% m/m)	2g:60ml	100–125°C	1 – 2h	Concentration: Glucose − 24.6 g/L Fructose − 9.7 g/L	Biofuel	[89]
Spent coffee grounds	Fatty acid methyl ester	H_2_SO_4_ (4% m/m)→ methanol →hexane	H_2_SO_4_ −1ml:8g	H_2_SO_4_ – n.d. Methanol − 55, 65, 75°C	H_2_SO_4_ − 3h Methanol − 4, 8, 12h	Efficiency: 90% (lipd)	Biofuel	[19]
Kans grass Sugarcane bagasse Wheat straw	Glucose Xylose	Inorganic acids (30% v/v) → H_2_SO_4_ Fermentation *Zymomonas mobilis Candida shehatae*	1g:10ml	100°C	45min	Maximum theoretical ethanol production: 82.45%	Biofuel - bioethanol	[86]
Municipal solid waste	Glucose	H_2_SO_4_ (0.5, 1% m/m)	1g:10g	130, 160°C	30, 60min	Concentration: Glucose − 43.2 g/L Ethanol efficiency: 44.6g/100g glucose	Biofuel – bioethanol	[90]
Oleaginous microalgae	Fermentable sugars	H_2_SO_4_ (3.5% m/v)	3g:97g	120°C	60min	Efficiency: Fermentable sugar - 256g/kg of biomass	Biofuel – bioethanol	[85]
Pineapple crown leaf	Cellulose nanocrystals	Pretreatment NaOH (5% m/m) NaOH (5% m/m) +H_2_O_2_ (16% v/v) Hydrolysis H_2_SO_4_ (60% m/m)	Pretreatment NaOH − 1g:20ml NaOH+H_2_O_2_ − 1g:40ml Hydrolysis H_2_SO_4_ − 1g:20ml	Pretreatment NaOH − 90°C NaOH+H_2_O_2_ − 55°C Hydrolysis H_2_SO_4_ − 45°C	Pretreatment NaOH − 1h NaOH+H_2_O_2_ − 90min Hydrolysis H_2_SO_4_ − 1h	Crystallinity index: 73%	Bio-polymer	[81]
Napier grass *Pennisetum purpureum*	Nanocellulose fibers	Pretreatment NaOH (12% m/v) Hydrolysis H_2_SO_4_ (20, 30% m/m) → water + zirconia balls	Pretreatment NaOH - n.d. Hydrolysis H_2_SO_4_ – n.d.	Pretreatment NaOH - n.d. Hydrolysis H_2_SO_4_ – n.d.	Pretreatment NaOH − 2h Hydrolysis H_2_SO_4_ – n.d.	Crystallinity index: H_2_SO_4_ (20%) − 60.0% H_2_SO_4_ (30% m/m) − 50.9%	Bio-polymer	[82]
Wool	Keratin Protein	NaOH (2% m/m)	10g:100ml	80°C	3h	Efficiency: Keratin: 25% Protein: 63%	Bio-polymer	[88]
Windmill palm raw materials	Nanocellulose fibers	NaOH (4% m/v), H_2_O_2_ (2% m/v); H_2_SO_4_ (64% m/m)	H_2_SO_4_ − 1g:15ml	NaOH, H_2_O_2_ − 85°C H_2_SO_4_ − 35°C	NaOH, H_2_O_2_ − 3h H_2_SO_4_ − 50 min	n.d.	Bio-polymer	[91]
Plant species *Agave americana L*. *Ricinus communis L.*	Cellulose nanocrystals	Pretreatment Toluene-ethanol (2:1 ratio) Alkaline Hydrolysis NaOH (15% m/m) →NaOH (3% m/m)+ H_2_O_2_ (2% m/m) + Na_2_SiO_3_ (3% m/m) H_2_SO_4_ (60% m/m) Acid Hydrolysis H_2_SO_4_ (40% m/m, 60% m/m)	Alkaline Hydrolysis NaOH − 1g:15ml NaOH+ H_2_O_2_ + Na_2_SiO_3_ − 1g:60ml → H_2_SO_4_ Acid Hydrolysis H_2_SO_4_ − 1g:40ml	Alkaline Hydrolysis NaOH − 60°C NaOH+ H_2_O_2_ + Na_2_SiO_3_ − 70°C H_2_SO_4_ − 45°C Acid Hydrolysis H_2_SO_4_ − 60, 45°C)	Toluene-ethanol − 8h Alkaline Hydrolysis NaOH − 1h NaOH+ H_2_O_2_ + Na_2_SiO_3_ − 1.5h H_2_SO_4_ − 2.5, 4.5h Acid Hydrolysis H_2_SO_4_	Efficiency: Alkaline hydrolysis − 94.1% Acid hydrolysis − 92.7%	Bio-polymer	[92]
Sugarcane bagasse	Nanocellulose fibers	Extraction NaOH (4% m/m)+H_2_O_2_ (24%) Hydrolysis Polypropylene+H_2_SO_4_ (0, 20, 40% m/m)+zirconia balls	Extraction n.d. Hydrolysis Polypropylene+H_2_SO_4_+ zirconia balls − 2g:160ml+200g	Extraction NaOH+ H_2_O_2_ − 60°C Hydrolysis Polypropylene+H_2_SO_4_ – n.d.	Extraction NaOH+ H_2_O_2_ − 2h Hydrolysis Polypropylene+H_2_SO_4_ − 12h	n.d.	Bio-polymer	[93]
Ginger fiber	Nanocellulose	Pretreatment Toluene+ethanol (2:1 ratio) NaOH (5% m/m) NaCO_2_+CH_3_COOH (4:1 ratio) Hydrolysis HCl (5M)	Pretreatment Toluene+ethanol - n.d. NaOH – n.d. NaCO_2_+CH_3_COOH – n.d. Hydrolysis HCl (5M)	Pretreatment Toluene+ethanol − 50°C NaOH − 50°C NaCO_2_+CH_3_COOH − 60°C Hydrolysis HCl − 50°C	Pretreatment Toluene+ethanol − 48h NaOH − 4h NaCO_2_+CH_3_COOH − 2h Hydrolysis HCl − 12h	Crystallinity index: 48%	Bio-polymer → Food packing	[94]
Bleached cellulose *Eucalyptus spp*.	Cellulose nanocrystals	H_2_SO_4_ (50% v/v)→ polypropylene → Ultrasound	1g:25ml	35, 40, 45°C	25, 30, 35min	Efficiency: 30.1–57.6%	Bio-polymer → Food packing	[95]
Vine shoots	Cellulose nanocrystals	Pretreatment NaOH (4% m/m) Acetate buffer + NaClO_2_ (0.315% m/m) Hydrolysis H_2_SO_4_ (64% m/m)	Pretreatment NaOH – n.d. Acetate buffer + NaClO_2_ – n.d. Hydrolysis H_2_SO_4_ – n.d.	Pretreatment NaOH − 80°C Acetate buffer + NaClO_2_ – n.d. Hydrolysis H_2_SO_4_ − 30min	Pretreatment NaOH − 2h Acetate buffer + NaClO_2_ – n.d. Hydrolysis H_2_SO_4_ − 50°C	Crystallinity index: 82%	Bio-polymer → nanocomposites materials	[96]
Brown, red, and green seaweeds	Nanocellulose fibers	Pretreatment HCl (0.2M) NaOH (4% m/m) KOH (5% m/m) NaOCl (6–10%) Hydrolysis H_2_SO_4_ (51% m/m)	HCl −1g:10ml NaOH − 1g:60ml →KOH→NaOCl − 1g:10ml H_2_SO_4_ − 1g:1g	HCl − 30°C NaOH − 75°C H_2_SO_4_ − 45°C	HCl − 2h NaOH − 3h KOH − 3h H_2_SO_4_ − 30 min	Efficiency: 21.5%	Bio-polymers Biomedical engineering, Wastewater treatment, Energy and electronics sector, Food packaging	[83]
Citrus processing waste	Essential oils and pectin	H_2_SO_4_ (0.75, 1.38, 2.0% m/m)→ hexane→ethanol	n.d.	100°C	15, 30, 45min	n.d.	Essential oils	[17]
Red seaweed *(Gracilaria birdiae)*	Cellobiose Glucose Galactose 5-HMF Levulinic acid Formic acid	H_2_SO_4_ (0.12–2.47M)	38.40–841.59g:1l	n.d.	3.26–36.73 min	Efficiency: Cellobiose − 5.1% Glucose − 4.6% Galactose 17.6% 5–HMF–3.9% Levulinic acid − 7.8% Formic acid − 5.4%	Fine chemicals Fermentable compounds	[97]
Shrimp Shell	Chitosan	Pretreatment HCl (1M) NaOH (3.5% m/m) NaOCl (0.315% m/m) Hydrolysis HCl (20, 25, 32, 37%) Microwave	Pretreatment HCl − 1:10 NaOH − 1:10 NaOCl – n.d. Hydrolysis HCl − 1:9 (m/v)	Pretreatment HCl − 75°C NaOH − 65°C NaOCl – n.d. Hydrolysis HCl − 90°C	Pretreatment HCl − 1h NaOH − 2h NaOCl − 5min Hydrolysis HCl − 90min	Efficiency: 70–85%	Glucosammine hydrochlorid	[98]
Porcine skin	Collagen	NaOH (3.5, 7% m/m)+NaCl (6% m/m)	n.d.	Freezing (−1.4, 1.6°C→ thawing	24h	n.d.	Meat jelly formulations	[87]
Triglycerides in milk	Fatty acids	NaOH (3M) →methanol →HCl	NaOH − 0.1g:2.5ml	NaOH − 80°C	NaOH − 15min	Efficiency: 70–91%	Medium for extraction of polycyclic aromatic hydrocarbons	[18]
Waste textiles	Glucose	H_2_SO_4_ (5, 32.5, 60% m/m)	40g:760g	30, 80, 130°C	1, 3.5, 6h	Efficiency: 70%	n.d.	[15]
Poultry feathers	Amino acids	supercritical water →semi-continuous flow-through subcritical reactor (5–10 ml/min)	1g:4-32g	210–250°C	n.d.	n.d. (identification of amino acids)	n.d.	[99]
Shrimp cephalothorax	Lipids Carotenoids	Water (pH 8.0) Alcalase (1.0% m/m) → Ultrasound	Water − 1g:1ml + alcalase	Water − 50°C →Alcalse − 60°C→ 90°C	Water − 3h → Alcalase − 2h → 15min	Efficiency: Lipids − 10-11g/100g Carotenoids − 8.6–8.8g/100g	n.d.	[100]

#### 4.2.1. Extraction of polysaccharides

Traditionally, both cellulose nanocrystals and nanofibers have been derived from wood. The new approach focuses on the search for alternative sources, with a particular focus on waste materials. Attempts have been made to isolate cellulose nanocrystals and nanofibers from pineapple crown leaves [81], windmill palm raw materials from windmill palms [91], sugarcane bagasse [93], ginger fibers [94], vine shoots [96], and brown, red, and green seaweeds [83]. The main directions of nanocellulose and nanofibers application include the production of highly specialized materials for medicine, food industry (bio-packaging) [94] and electronics [83]. Despite the different compositions and properties of the proposed raw materials, the production of cellulose nanocrystals and nanofibers involves three main steps: (1) pretreatment, (2) bleaching, (3) acid/alkaline hydrolysis. Pretreatment (e.g. extraction) uses a sodium hydroxide solution of 4 to 12% [81,82,93] or organic substances (toluene/ethanol) [94,101]. For the bleaching step, sodium hypochlorite [83] or sodium chlorite [96] is used. The studies also present the possibility of conducting the process with additional components such as hydrogen peroxide or sodium silicate [92]. An essential unit process is acid or alkaline hydrolysis. The process is carried out with sulfuric acid [81], hydrochloric acid [94] or sodium hydroxide. Evdokimova et al. (2021) first used *Agave americana L*. and *Ricinus communis L*. fibers as sources for the production of cellulose nanofibers. Acid hydrolysis using sulfuric acid and alkaline hydrolysis using sodium hydroxide was employed for the process. Highly crystalline cellulose nanofibers with a crystallinity index of 94.1% and 92.7% were obtained for both the alkaline and acid treatment steps.

Process control parameters have an extremely significant impact on both process efficiency and product quality. For example, reaction temperature, time, and acid type can change the crystallinity and morphology of nanocrystal cellulose, including rod, sphere, and needle shapes [96]. The research focuses on hydrolysis with sulfuric acid over a wide range of concentrations from 20 to 64% (Table 2). The contact time of the solid phase with the hydrolyzing medium ranges from 30 min to 12 h, at temperatures of 35 to 50°C. A higher process temperature could affect the breakdown to simple sugars. A correlation is observed that contact time should be increased and the hydrolysis temperature should be higher when acids with low concentrations are used. The use of sulfuric acid at a lower concentration has been shown to have a much better effect on the quality of isolated cellulose nanocrystals [82].

#### 4.2.2. Extraction of simple sugars and lipids – production of biofuels

Because of their high lignocellulose, starch, protein, or pectin content, waste materials have a high potential for biofuel production. Most biodegradable wastes can be digested and converted to ethanol and biogas. Biomaterials from waste can be converted to biogas through the following steps: hydrolysis, acidogenesis, acetogenesis, and methanogenesis [90]. In studies, orange peel waste [89], Kans grass, sugarcane bagasse, wheat straw [86], municipal solid waste [90] or oleaginous microalgae [85] were hydrolyzed to simple sugars. The isolated sugars are used for fermentation processes. Hydrolysis of waste materials into simple sugars is carried out in an acidic environment. The primary medium used for the process is sulfuric acid with concentrations ranging from 0.5 to 3.5% (Table 2). For the decomposition to monosaccharides, high process temperatures (>100) can be used, reaching up to 160°C [90]. Such temperatures allow contact time to be reduced in the range of 0.5 to 2 h.

Oily microalgae have more than 20% lipids in their dry weight and have been considered a potential biodiesel material. Plants also have carbohydrates that can be a source of simple sugars. Mandik et al. (2020) [85] proposed a method to convert algae to simple sugars. Chloroform and methanol were used as cosolvents used for extraction. Hydrolysis of simple sugars was carried out in sulfuric acid (3.5% m/v) at 120°C for 60 min. The efficiency of the process was 44.8 g of fermentable sugar/kg of biomass [85]. The sugar-rich hydrolyzate is a precious raw material for biofuel production, but the hydrolysis residue, which contains a significant amount of lipids (for selected materials), is equally valuable. Lipid extraction is carried out using hexane as a solvent. Studies have shown that the use of this type of organic liquid allows the extraction of lipids at a level of 90%. The proposed method allows the selective separation of lipids and fermentable sugars, making the method waste-free [19]. Waste materials rich in lipids and carbohydrates represent considerable potential for biofuel production. Proper selection of unit processes allows selective separation of monosaccharides, which can be used in fermentation processes, and lipids, which are feedstock in biodiesel production. Adequate cycling may allow the development of waste-free biofuel production technologies.

### 4.3. Enzymatic hydrolysis

Enzymatic hydrolysis is the main tool of scientists in the field of amino acid extraction from high-protein waste. The most commonly produced hydrolyzates are used as food supplements, additives, or animal feed. Information about hydrolyzate application on plants is strongly limited despite their rich amino acid profile, which is crucial for plant development, and high content of macroelements such as phosphorus. It is also worth mentioning that it is possible to hydrolyze the polyvinyl acetate by using Ophiostoma, widening range of applications for this process [102]. Enzymatic hydrolysis is also implement as a pretreatment to improve the release of bacterial proteins e.g. E. coli, to obtain greater L-tryptophan production through fermentation [103]. The wide range of applications and products makes the optimization of process parameters necessary (Table 3).

**Table 3.** Enzymatic hydrolysis of waste materials as a valorization process to useful products.

Material	Product	Medium	pH	Temperature	Time	Effect	Application	References
Shrimp shell	Protein, chitin	Aspartic proteases P6281; saccharopepsin	Aspartic proteases P6281: 3.0 Saccharopepsin: 5.0	40°C	6h	Recovery rate: Protein: 91.4% Chitin: 88.9%	Anti-inflammatory and antioxidant agents	[104]
Lignocellulosic biomass	Hexose, pentose	ethanologenic recombinant E. coli strain (FBr5)	6.0	35°C	104h	45 g of ethanol per L	Ethanol	[101]
Shrimp waste	Amino acids	Alcalase	7.87	58.87°C	67.76min	Degree of hydrolysis 27.90%	Feed for aquaculture	[105]
Food waste	Amino acids	15.5% pepF and 84.5% pepA	3.0–5.0	n.d.	60h	Yield: 23.9 mg/mL	Fertilizer	[7]
Whey	Amino acids, peptides	Papain; pepsin	n.d.	37°C − 42°C	8h	Degree of hydrolysis: 48%	Fertilizer	[106]
Muscles of Acipenser sinensis	High protein hydrolyzate	papain	6.0	70°C	6h	17.47% the yield of protein hydrolyzate	Food	[107]
Oncorhynchus mykiss	Amino acids	Alcalase and papain	Alcalase: 8.0; Pepsin: 2.0	37°C	120min	Degree of hydrolysis: Alcalase: 44.08%; Papain: 27.62% Amount of amino acids: Alcalase: 33.53%; Papain: 29.39%	Food additives	[108]
Engraulisencrasicolus	Amino acids	Protamex; Flavourzyme; Alcalase	7.5	50°C	3h	n.d.	Food additives	[109]
Sardine solid waste	Amino acids	pepsin	1.5	48°C	4h	Yield: 50 $\frac{g}{g\cdot mg}$	Food additives	[110]
Fleshing meat	Amino acids	Alcalase and papain	Alcalase: 6.5; Papain: 7.5	60°C	n.d.	Solubilization: 97%	Gelatin production, thickening agents, texturizers	[111]
Bacterial cell	L-tryptophan	trypsin	8.0	37°C	6h	53.87 g/L L-tryptophan production	L-tryptophan	[103]
Polyvinyl acetate	Polyvinyl alcohol	Ophiostoma piceae	7.0	28°C	48h	n.d.	Recycled paper	[102]
Lignocellulosic biomass	Reducing sugar	Cellic Ctec2 cel-lulase	5.5	55°C	24h	n.d.	Reducing sugar	[112]

A very important aspect is the determination of optimal conditions of hydrolysis. The evaluation of pH, temperature and incubation time were conducted in work by Noman et al., (2018) [107], The muscles of the Chinese sturgeon (*Acipenser Sinensis*), were subjected to papain. The efficiency of the hydrolyzate was shown at 17.47%, with the degree of hydrolysis of 24.89%, wherein the highest amino acid content was 96.35%. It was indicated that extending the incubation time and solid to liquid ratio (1:1 optimal) negatively affects the efficiency of the process. The influence of the enzyme concentration was also considered, where low efficiency was obtained at low values and at higher values loss of efficiency was noticed. Based on research, it was found that the concentration of papain at the level of 3% is the optimal solution. In the case of pH, it is that a greater degree of amino acid recovery was obtained around the neutral pH due to the denaturation of enzyme proteins. The effect of temperature indicated that using too low temperature (<70°C) results in a lower degree of hydrolysis. However, as in the case of the enzyme concentration, too high a value leads to worse results>75°C. It has been suggested that this phenomenon is due to the denaturation of enzymes as in the case of pH. A similar evaluation with the Response Surface Methodology was conducted in the article by Nguyen et al., (2021) [105] on the management of shrimp waste with alcalase. Under comparable conditions, a 27.90% degree of hydrolysis was obtained. The parameters were tested in narrow ranges for pH (7–9), temperature (50–70°C), time (50-70 min), and an enzyme to substrate ratio (0.1–0.25), where the optimal values were respectively 7.87; 58.87°C; 67.76 min; 0.24%. The justification for pH tendency similarity follows from the range of enzyme activity within a given pH range, where exceeding that range results in a reduction in activity. As for the study of the optimal parameters for hydrolysis, a combination of modeling and sensitivity analysis was applied, which allowed for more precise statistical data [113]. This procedure was has been carried out for sugar extraction through cellulase, but applying it to amino acid extraction may result in more accurate correlations between the effects of each parameter.

The above publication evaluated the applicability of various enzymes that indicated similar conditions for hydrolysis. In work by Anzani et al., (2018) [111], pepsin, pancreatin, trypsin, alcalase, papain, and dispase were tested on subcutaneous meat of the bovine animal. The highest degree of solubilization (97%) was demonstrated for alcalase and papain, whereas for the remaining enzymes the efficiency was a maximum of about 38%. The parameters of hydrolysis for alcalase and papain were pH 6.5, in the presence of Na_2_HPO_4_ 10 mM and 7.5 for Na_2_HPO_4_ 10 mM, EDTA 2 mM, dl-cystine 4 mM, respectively, at 60°C. The authors suggested that these enzymes are the most effective means of effective extraction of amino acids. The usage of alcalase is more indicated due to a much higher degree of hydrolysis of 30.9% compared to 6.5% for papain and a lower cost of this enzyme. Another comparison of the functionality of enzymatic hydrolysis with 2% alcalase and 2% pepsin was conducted on a rainbow trout (*Oncorhynchus mykiss*) [108]. It was shown that the degree of hydrolysis was 16.46% higher in the case of pepsin, but the amino acid content in the alcalase-based hydrolyzate was higher by 4.14%. Both hydrolyzates presented emulsifying and antioxidant properties, which allows them to be used as food additives.

The above research indicated the usage of single enzymes for enzymatic hydrolysis nonetheless, the use of proteases mixture was also considered. With the help of protamex, flavourzyme, and alcalase, the ratio was 1.1:1.0:0.9, respectively, with the enzyme concentration of 3%. The resulting product from Anchovies (*Engraulis encrasicolus*) was spray-dried and dedicated as a feed or food additive with 42% of total amino acid content [109]. The combination of various proteases along with chitinase and ethyl acetate has been evaluated by Deng et al., (2020) [104], where shrimp shell waste, rich in chitin and proteins, astaxanthin was subjected to hydrolysis. Shrimp wastes are particular due to their high methionine content, which requires extraction. The recovery of this amino acid is particularly important because it is the only one, apart from cysteine, amino acid to contain a sulfur atom in the structure. This allows for the formation of other components in the body containing sulfur molecules and initiates the production of proteins [114]. The recovery method is crucial considering the tendency in the production of seafood and the quantity of shrimp that end up as waste and is not utilized in for further applications, despite its rich composition. To extract the amino acids, alcalase and papain were reused, this allowed for 91.4% amino acids recovery with antioxidant properties. The above research indicates that the most effective enzymes are alcalase and papain, with similar hydrolysis conditions, what allows for their simultaneous application one after another, reducing the need for equipment or process parameter changes. Albeit in the case of each medium and the extraction of specific amino acids, precise optimization for a particular enzyme is of key importance, but when it comes to the parameters of the process, they depend mainly on a given enzyme rather than the material on which the process is carried out.

The main problem with the utilization of enzymatic hydrolysis is low profitability, therefore, it is proposed to use a range of pretreatment processes that will allow the preparation of material for amino acid extrusion. Application of homogenization, ultrasound, microwaves, and pulsed electric fields are considered to promote protein unfolding [115]. Although depending on the purpose of given hydrolysis, different pretreatment should be considered. For obtaining thermally stable proteins, thermal treatment should be used to partially denaturalize the proteins, shortening the extraction time. The influence of ultrasounds on the extraction of free fatty acids was verified in the work by Huang et al., (2020) [10], where an increase in efficiency of 18.9% was noted during 5 min of process conducting. The effect of ultrasound on amino acid extractions was verified by Lan et al., (2020) [116], where it was found that it is possible to shorten the hydrolysis time of pig skins by 3 h, with an increase in the amino acid content by 15.98% and the degree of hydrolysis by 4%. The optimization of a given solution was performed, wherein the influence of individual parameters on the degree of hydrolysis was established, frequency (optimal combination 20, 40, 40), power (600 W), and time (25 min). In the case of sugar extraction, the influence of surfactants was investigated. It was indicated that their effect depends purely on the type of substrate on which they were used. The addition of a surfactant tends to create more reaction sites for the inclusion of individual enzymes [112]. Investigating this in the case of amino acid extraction is one of the development pathways worth considering. In the case of biogas production, attempts were made to utilize freezing-thawing pretreatment and microbial electrolysis cell, which presented an increase of up to 70%. It has also been shown to increase the degree of solubilization of aromatic proteins, which may translate into the degree of amino acid extraction during the application of proteases [117].

It is suggested to introduce pretreatment to limit the parameters of the process and increase the availability of individual proteins through their partial degradation, allowing for a precise determination of the potential of enzymatic hydrolysis in the extraction of amino acid for a given medium. Because the treatment with ultrasounds, microwaves, and temperature, which allows for increasing the degree of solubilization and the availability of certain groups of amino acids. As a result, it will be possible to determine the optimal group of enzymes for extracting individual amino acids.

### 4.4. Thermal treatments in the extraction of amino acids

Enzymatic hydrolysis is the most effective method for the extraction of amino acids. Even so, other types of hydrolysis are being researched in this field. One of them is thermal hydrolysis, it has been indicated that it is possible to completely solubilize food waste, obtaining about 0.5% of amino acids. Optimization for the temperature was applied, where 180°C allowed the solubilization of nutrients at the level of 80% K, 95% P and 50% N [118]. Fertilizers made by thermal methods showed a positive effect on plants [119]. However this technology, requires elevated energy levels inputs to ensure appropriate process conditions. In addition, the action of such high temperature causes the degradation of valuable amino acids, which are necessary to achieve the biostimulating effect [120]. The usage of subcritical hydrothermal treatment was also investigated, where three process parameters were optimized (temperature, pH, reaction time). It was found that the action of alkali showed the highest degree of nutrient solubilization, with a temperature of 180°C and 1.5 h, 82.6% recovery of nutrients was retrieved from organic waste such as chicken manure and peanut meal [121]. The main problem of the method is the high operation cost along with the necessity to maintain a subcritical water state, requiring high pressure. The mentioned technology is advantageous for the recovery of valuable nutrients without the excessive extraction of heavy metals. Scallop entrails contain significant cadmium content, which is difficult for proper management. Although by utilizing subcritical treatment (200°C, 20 min), almost complete degradation of the material can be achieved, without extraction of heavy metals [122].

### 4.5. Comparison of chemical and enzymatic hydrolysis

**Figure 3.**
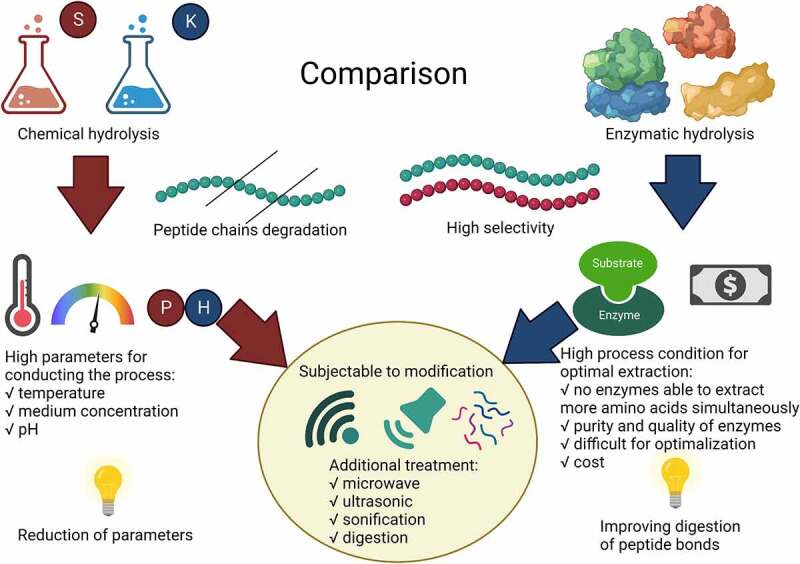
Comparison of enzymatic and chemical hydrolysis in terms of amino acid extraction.

Some studies show that chemical hydrolysis is ineffective in extracting amino acids as a result of the significant degradation of the peptide chains. The main problem in using chemical hydrolysis is the degradation of peptide chains at low pH (<2) or high pH (>9). As a result of these conditions, tryptophan is degraded [20]. Other amino acids such as aspartic acid, glutamic acid, tryptophan and isoleucine showed degradation by alkaline hydrolysis of KOH, NaOH up to 150°C [123]. Instead, enzymatic hydrolysis is a widely utilized method that allows for the precise recovery of particular amino acid groups. When amino acids to stimulate plants, the main issue is determining the amino acid profile of the biostimulants obtained. Chemical hydrolysis may cause degradation of amino acids and result in a low degree of their recovery, while the use in this case of pepsin allowed obtaining a significant amount of amino acids [20,110,124]. Enzymatic hydrolysis has been established as an effective method for the recovery of amino acids without considerable damage to their structure, as a paper by Sun et al., (2019) [7], has shown that it is possible to achieve up to 51.2% protein recovery efficiency using a combination of enzymes PepF and PepA. This indicates the potential of enzymatic hydrolysis in precise amino acid recovery and the formation of bio-fertilizers [106]. In the work by Trakselyte-Rupsiene et al., (2021) [125] it was determined that enzymatic hydrolysis, despite its high costs and the need to use high purity reagents, can be modified to support amino acid extraction through the utilization of sonication. The method of pretreatment of the material supports the extraction of amino acids that are valuable to plants, as shown in tests on wheat grain seeds. The use of ultrasound pretreatment resulted in a 2.2-fold increase in the free amino acid profile, which resulted in the stimulation of plant growth. The plant yields were 30.1% and 33.8% for the root length and stem length, respectively. Poultry waste containing large amounts of creatine undergoes chemical hydrolysis for amino acid degradation, mainly loss of tryptophan. The necessity of using heavy extraction conditions, including high temperature and CO and SO_2_ emissions to the environment, suggests that this is not a suitable method for waste management in terms of creatine extraction [14]. It is also noted that enzymes and ionic liquids are more environmentally friendly. However, chemical hydrolysis is a highly efficient process with low costs than other extraction methods and constant yield independent of many process factors [14]. It is worth noting here that Izydorczyk et al., (2022) [13] evaluated that the chemical hydrolysis process can be an excellent disposal method, particularly for waste of various origins, for example, poultry waste. It should be noted that enzymatic hydrolysis prevents racemization of amino acids, vitamins, oligosaccharides and tryptophan, thus being highly influenced by temperature and acid and alkali treatment [126]. The article by Colantoni et al. (2017) [47] verified the differences in CO_2_ emissions between enzymatic and chemical hydrolysis, where it amounted to 57%, and the energy consumption needed to obtain fertilizer by 26% in favor of the use of enzymes. This presents the advantage of enzymatic hydrolysis in terms of environmental impact. Enzymatic hydrolysis is the leading way to obtain a high recovery efficiency of proteins with a low molecular weight, which allows them to be better absorbed by plants [127]. The main issue of the large-scale application of enzymatic hydrolysis is the need to adapt the extraction equipment. This requires optimizing the infrastructure and the consumption of enzymes for a given material, which makes this method expensive. Although enzymatic hydrolysis is a process that can be carried out at low concentrations and any liquid-to-solid ratios, thus indicates the flexibility of the process parameters. The chemical composition itself depends on the type of enzyme utilized, therefore, it is crucial to study the exact structure of the obtained for fertilization purposes [128]. A direct comparison of the two processes is shown in Figure 3.

### 4.6. Chemical hydrolysis in agriculture

Hydrolysis allows additional elements to be introduced into the solution from the medium involved in the process. Solid and liquid phases have fertilizing properties since macronutrient transfer occurs in both phases, often in different proportions. The main reason for using KOH is to increase the potassium content required for the proper development of the plant. Alkaline hydrolysis is often used to manage livestock waste, such as dead animals and infected animal carcasses, mainly because of its ability to eliminate pathogens and extract nutrients. However, an important issue is the incorporation of additional nutrients into the formulation, as most of the resulting fertilizers are unbalanced in terms of N to P ratios [129]. The study by Akdeniz and Yi (2021) [130] investigated the possibility of using hydrolysis without manipulating pressure and temperature. It was shown that after 31 days of exposure to waste, all potential pathogens were removed and an increase in fresh plant weight of 18.2% was achieved. Part of the work has indicated that the use of auxiliary processes in the hydrolysis process to extract amino acids can positively affect the nutritional composition and amino acid profile. The article by Bhat et al., (2021) [12] presented that the use of the acoustic and hydrodynamic cavitation process allows the generation of more amino acids with higher nutritional value through the degradation of disulfide bonds in the case of proteins, contained in hair waste. As a consequence of the mentioned process, significant amounts of ornithine, cysteine, taurine, and proline were obtained compared to ultrasonic and non-ultrasonic treatment. In the work by Holkar et al., (2016) [131], alkaline hydrolysis of 3% KOH and 0.24% NaOH was applied, where the acoustic cavitation process allows the reduction of the process conducting. The extraction was carried out at room temperature and low pressure using the 20 kHz sonicator, allowing the hydrolyzate to be obtained, which influenced the dry weight of the plants, increasing them by 84% compared to the control sample prepared without cavitation. The use of microwaves as a pretreatment to stimulate alkaline hydrolysis was also investigated. The utilization of microwaves at 2.45 GHz, 643 W, and 10% intensity improved the degree of degradation of the shrimp exoskeleton degradation. As a result of microwave activity, the amount of sucrose and glucose sugars in the liquid phase increased than in the solid phase. The main effect was the higher solubilization of the complex chars [132]. Such studies present that, although chemical hydrolysis destroys some amino acids, the use of auxiliary processes and the optimization of the extraction process allow the method to be utilized as a cheap and simple alternative to the disposal of high-protein waste. The use of other media for the alkaline hydrolysis process in addition to KOH and NaOH has also been verified. When sewage sludge management was attempted, the best results of organic nitrogen recovery were obtained for Ca(OH)_2_. Where nitrogen solubilization in the fertilizer obtained was 54% and organic N maintenance>80% [133], indicating the possibility of using chemical hydrolysis to reduce waste of various origins, where adaptation of the extraction medium seems to be a significant aspect. The alkaline hydrolysis process has been identified as an interesting way of dealing with dead slaughtered animals that cannot be used for further processing due to infection or diseases. The work of Kang et al., (2019) [129] indicated that the usage of 90% KOH at a temperature of 100°C is able to obtain liquid fertilizer from slaughter waste without removing more heavy metals harmful to the environment. It should be noted that the obtained fertilizer is not a good substitute for artificial fertilizers, mainly because of the lack of an appropriate N/P ratio, but it has been shown to supplement fertilization with mineral ones. It is also possible to manage industrial waste such as combustible cartridge cases, which is a constantly occurring waste in the modern world. KOH was used with a concentration of 85% at a temperature of 70°C, which allowed up to 87% nitrogen recovery. The verification of the fertilizer obtained on plants indicated an overall growth in biomass of 938% compared to the control, thus a phenomenal result for a 100% dose. The tendency was also occurring in the shoots, a rise of 109.6% for the same dose, although the chlorophyll content decreased by 17.6%. Only the 25% dose allowed for the increase of all important plant growth parameters [134]. The mentioned method allows for easy and cheap disposal of this type of waste without emitting 100% of the nitrogen contained in it into the atmosphere. This points to the possibility of using chemical hydrolysis in a wide range of industrial waste, not just purely organic.

The paper by Massaya et al. (2022) [126] showed that spent coffee grounds could be managed by chemical and enzymatic hydrolysis. The possibility of complete management of all streams was indicated, including the solid phase after hydrolysis, subjected to hydrothermal conversion. Primary chars were obtained and their use on plants increased by 35% in root length. This approach indicates that it is possible to create precipitation-free valorization processes in which the material is fully utilized. The work of Gaidau et al. (2021) [135] describes the possibilities of using low quality wool for fertilization purposes. Enzymatic hydrolysis was applied, where proteases were indicated [70], thus allowing the secretion of creatine with a lower molar mass and obtaining a large amount of bioactive peptides. Alkaline hydrolysis has been determined to be a highly efficient and easy-to-apply method for the recovery of creatine recovery from waste materials and is the basis for further modifications, e.g. with enzymes. This demonstrates the potential of combined methods for amino acid recovery, where chemical hydrolysis is the medium for the initial cleavage of peptide bonds. The action of superheated water hydrolysis (140°C; 170°C) was compared to conventional alkaline hydrolysis performed with KOH (5–15%; 140°C; 170°C). Both methods resulted in low molecular weight amino acids. Alkaline hydrolysis showed a higher degree of extraction of the following amino acids Glu (13.03%); Leu (51.76%); Asp (71.29%) compared to superheated water hydrolysis. Although the mentioned method preserves more of the amino acids such as Ser; Thr and reduces the harmfulness of the amino acid extraction process [21]. The major problem of utilizing chemical hydrolysis is the negative impact on the environment and the degradation of some amino acid groups. The creatine obtained as a result of hydrolysis processes has a positive effect on plants at the dose of 5% hydrolyzate, which caused an increase in the increase of wheat stem of 10.7% and 18.3% depending on the type of plant. The extraction was carried out using alkaline hydrolysis with KOH, where the highest degree of creatine extraction (87.10%) was achieved for KOH at 3% and 75°C. The utilization of higher concentrations and temperatures has been shown to lead to the degradation of creatine and a reduction in efficiency to 65.54% [124]. The creatine distribution was confirmed in the work of Gousterova et al. (2014) [136] using a mixture of KOH and NaOH at 120°C and a pressure of 2.03 atm, about 20% was only insoluble, partially degraded creatine. Research was also carried out on the management of collagen-rich tanning industry waste to create fertilizer compositions. The use of mixed extraction media was also verified by Nustorova et al. (2005) [137], where 0.15 M KOH and 0.05 M NaOH were used at 120°C. 82% of wool solubilization was obtained, with 90.3% of proteins and the liquid phase. Although, as in previous studies, 20–25% was partially degraded keratin, which indicates the need for further optimization to completely degrade the keratin material completely. The fertilizer obtained improved the germination degree of plant germination from 40% to 80%, additionally, the hydrolyzate turned out to be an effective agent to cultivate microorganisms positive for plant development (i.e. *mixotrophic bacteria, Actinomycetes, Microomycetes Baciullus Mycoides)*. This indicates the possibility of using hydrolyzates as a medium used to culture microorganisms and subsequent application to the plant root system for faster assimilation and development. In the article by Zainescu et al., (2016) [138] acid hydrolysis in H_2_SO_4_ (1.3–2.8%) with the addition of corn starch and dipotassium phosphate, which act as additives to support plant growth. The hydrolyzate obtained was then added to the hydrogels, creating a fertilizer composition with a maximum total nitrogen content of 13.71% and proteins at the level of 77.05%. This shows the high efficiency of the modified acid hydrolysis process under minimally invasive conditions. The work itself did not conduct a direct evaluation of products on plants, this is an issue for future research.

## 5. Future perspectives and guidelines

**Figure 4.**
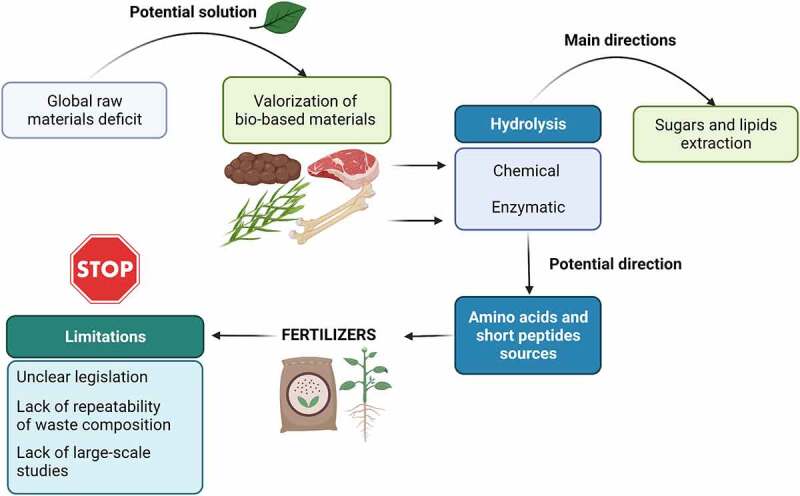
Directions for valorization of bio-waste and limitations in technology implementation.

In light of the global deficit of many raw materials, the valorization of bio-based materials might be a likely and needed development direction. One of the main methods of waste treatment, next to enzymatic processes, is chemical hydrolysis. The literature mainly focuses on the possibility of extraction of sugars, lipids, or to a much lesser extent, amino acids and short peptides (Figure 4.).

The lines of application of the isolated components are largely monothematic (production of composite materials or production of biofuels). Chemical hydrolysis is mainly performed on plant biomass and to a lower level on animal waste. Particularly noteworthy are high-protein materials, which are a source of amino acids valuable for fertilizer. The process of chemical hydrolysis, under appropriate conditions, is a very good way to break down proteins into amino acids and short peptides. The increasing demand for food and the current geopolitical situation are forcing the identification of alternative sources of fertilizer nutrients and the development of formulations to increase crop yields. There are a few reports on the use of chemical hydrolysis as a unitary process in the production of plant growth biostimulants. Animal wastes appear to be attractive high-protein materials, but the most problematic ones cannot be processed for fertilizer purposes due to limiting legislation. These materials are considered disease carriers, often with high infectious potential. An additional limiting element is fertilizer quality requirements. The barrier could be overcome by individual assessment of the applicability of a given material in a specific management direction, supported by many studies confirming its usefulness and safety.

An analysis of the literature indicates that there are no general guidelines for hydrolysis process parameters that are reproducible when processing seemingly similar materials. Authors often forget about a very important aspect, which is the cost-effectiveness of the proposed process. A basic economic analysis of the solution should be an integral part of this type of work. The problem limiting the implementation of the technology is the frequent lack of repeatability of waste composition, which is unattractive for producers and requires a lot of expenses. Proposals that allow the recovery of a valuable raw material while producing another waste with no indication of how it will be managed are also not very interesting to the industry. The chemical hydrolysis process has been widely studied and is a unitary process that allows the valorization of waste for various purposes. Only few works focus on scaling up the developed technology in the literature, ending the research at the laboratory stage. It is worth noting that both acid and alkaline hydrolysis can proceed quite differently on a small scale versus a technical or semi-technical scale. The key to success could be the development of a universal installation allowing for the flexible change of process conditions depending on the waste processed and the expected final product. This would require performing a number of process parameter optimizations for each group of renewable raw materials.

## 6. Conclusions

As a result of the literature analysis, it was found that the enzymatic hydrolysis process is much more efficient in terms of amino acid extraction compared to chemical hydrolysis. The reason is the too severe degradation of the peptide bonds of the proteins due to the acting of high concentration of the extraction media and temperature. Enzymatic hydrolysis through a wide range of different enzymes allows for the precise recovery of individual amino acids. However, chemical hydrolysis is a process that is susceptible to modifications, such as sonication, treatment with ultrasound, or indirect processes, which prepare the material for extraction. The mentioned property allows reducing the negative features of chemical hydrolysis, i.e. protein degradation, high operating costs, or greenhouse gas emissions, due to the basement of the severity of the process. The introduction of optimization and indirect processes to improve the chemical hydrolysis for the extraction of amino acids seems to be the way for future research. It is especially recommended due to the high availability of extraction media, the possibility of modification of the process, and enriching the composition at the production stage.
